# Enhancement of Electricity Production by Graphene Oxide in Soil Microbial Fuel Cells and Plant Microbial Fuel Cells

**DOI:** 10.3389/fbioe.2015.00042

**Published:** 2015-04-01

**Authors:** Yuko Goto, Naoko Yoshida, Yuto Umeyama, Takeshi Yamada, Ryugo Tero, Akira Hiraishi

**Affiliations:** ^1^Electronics-Inspired Interdisciplinary Research Institute (EIIRIS), Toyohashi University of Technology, Toyohashi, Aichi, Japan; ^2^Department of Biomedical Science, College of Life and Health Science, Chubu University, Kasugai, Aichi, Japan; ^3^Center for Fostering Young and Innovative Researchers, Nagoya Institute of Technology, Nagoya, Aichi, Japan; ^4^Department of Environmental and Life Sciences, Toyohashi University of Technology, Toyohashi, Aichi, Japan

**Keywords:** soil microbial fuel cell, plant microbial fuel cell, graphene, graphene oxide, extracellular electron transfer

## Abstract

The effects of graphene oxide (GO) on electricity generation in soil microbial fuel cells (SMFCs) and plant microbial fuel cell (PMFCs) were investigated. GO at concentrations ranging from 0 to 1.9 g⋅kg^−1^ was added to soil and reduced for 10 days under anaerobic incubation. All SMFCs (GO-SMFCs) utilizing the soils incubated with GO produced electricity at a greater rate and in higher quantities than the SMFCs which did not contain GO. In fed-batch operations, the overall average electricity generation in GO-SMFCs containing 1.0 g⋅kg^−1^ of GO was 40 ± 19 mW⋅m^−2^, which was significantly higher than the value of 6.6 ± 8.9 mW⋅m^−2^ generated from GO-free SMFCs (*p* < 0.05). The increase in catalytic current at the oxidative potential was observed by cyclic voltammetry (CV) for GO-SMFC, with the CV curve suggesting the enhancement of electron transfer from oxidation of organic substances in the soil by the reduced form of GO. The GO-containing PMFC also displayed a greater generation of electricity compared to the PMFC with no added GO, with GO-PMFC producing 49 mW⋅m^−2^ of electricity after 27 days of operation. Collectively, this study demonstrates that GO added to soil can be microbially reduced in soil, and facilitates electron transfer to the anode in both SMFCs and PMFCs.

## Introduction

The microbial fuel cell (MFC), a system that converts chemical energy into electrical energy using microorganisms, has received a growing amount of attention as a technology for recovering energy from organic residues (Logan et al., 2006). In addition to the MFCs that use microbial cultures, there are several types of sediment, or soil microbial fuel cells (SMFCs), which typically consist of sediment or wet soil sandwiched between two electrodes. These electrodes are placed on the bottom of the sediment (anode) and either on top of the sediment or suspended in the water phase (cathode) (Reimers et al., 2001; Tender et al., 2002). In some studies, electrons for the MFC were donated from organic matter excreted from plants, which is known as the plant microbial fuel cell (PMFC) (De Schamphelaire et al., 2008; Kaku et al., 2008; Strik et al., 2008). SMFCs and PMFCs have great promise, not only for sustainable electricity recovery from the environment, but also for their potential application in supplying electricity in self-powered devices. Such applications include devices that monitor environmental parameters (Donovan et al., 2008), sensors to monitor the maturity of plants (Chen et al., 2012), and applications in bioremediation, and recovery of heavy metals from contaminated environments (Gregory and Lovley, 2005).

The electrical productivity in SMFCs and PMFCs is affected by a number of factors, such as the speed of substrate oxidation, electron transfer to the anode, proton transfer from the anode to the cathode, and oxygen reduction at the cathode. A number of modifications and different conditions have been investigated to improve the productivity of these systems in SMFCs and PMFCs (Rezaei et al., 2007; Strik et al., 2008; Nielsen et al., 2009; Helder et al., 2010, 2012; Takanezawa et al., 2010; Timmers et al., 2010). Both the quality and quantity of microbial populations developed in SMFCs and PMFCs directly affect the electrical productivity of the system (Kaku et al., 2008; De Schamphelaire et al., 2010; Pisciotta et al., 2012). Modification of the anode is also one of the most important strategies for improving the performance of SMFCs and PMFCs. Several materials have been used to increase the surface area to allow for the attachment of a greater number of microorganisms. These materials include graphite felt, graphite granules (Timmers et al., 2013), Biochar (Huggins et al., 2014; Lu et al., 2014), active carbon (Song et al., 2012; Karra et al., 2014), and graphite grains (Helder et al., 2010).

It has recently been shown that members of the genus *Shewanella*, well known as electricity-producing bacteria, are capable of reducing graphene oxide (GO) to electrically conductive graphene (Salas et al., 2012). GO is an intermediate product of graphene, obtained by chemical exfoliation of graphite. Microbial GO reduction took place via respiratory extracellular electron transfer (EET), where GO was used as the terminal electron acceptor (Salas et al., 2012). It has been shown that GO can enrich bacteria capable of EET and the resultant reduced form of GO functions as an electrode with the attached bacteria. The addition of GO was found to facilitate electricity production at both the anode and the cathode in MFC when microbial cultures were used (Yuan et al., 2012; Zhuang et al., 2012).

Graphene oxide has also received considerable attention as an absorbent of a number of toxic chemicals, including heavy metals and organic pollutants (Chowdhury and Balasubramanian, 2014). On the other hand, both GO and graphene have recently been reported to have toxic effects on a number of living systems (Seabra et al., 2014). Nevertheless, the potential ecological risk of GO when applied to SMFCs and PMFCs may be reduced by assuming that GO-used systems can control the redox state of heavy metals as pollutants, thereby decreasing the uptake of toxic metals by plants.

In this study, we examine the effects of the addition of GO on electricity generation in SMFCs and PMFCs. First, SMFC with different concentrations of GO was tested for generating electricity. Second, the concentration of GO which resulted in the highest amount of generated electricity in the SMFC was applied to PMFCs to evaluate its effects on electricity generation and plant growth.

## Materials and Methods

### Preparation of GO

Graphene oxide from graphite powder with 30-μm particle size (SEC Carbon, Kyoto, Japan) was prepared according to the modified Hummer’s method (Tung et al., 2008), with modifications as reported previously (Tanizawa et al., 2012). Approximately, 7.9 g (dry wt.) of GO was obtained from 10 g of graphite powder. The prepared GO was dissolved in sterilized MILLIQ^®^ water to a concentration of 10 g⋅L^−1^ and stored at 4°C until use.

### SMFC construction and operation

**Figure 1:**
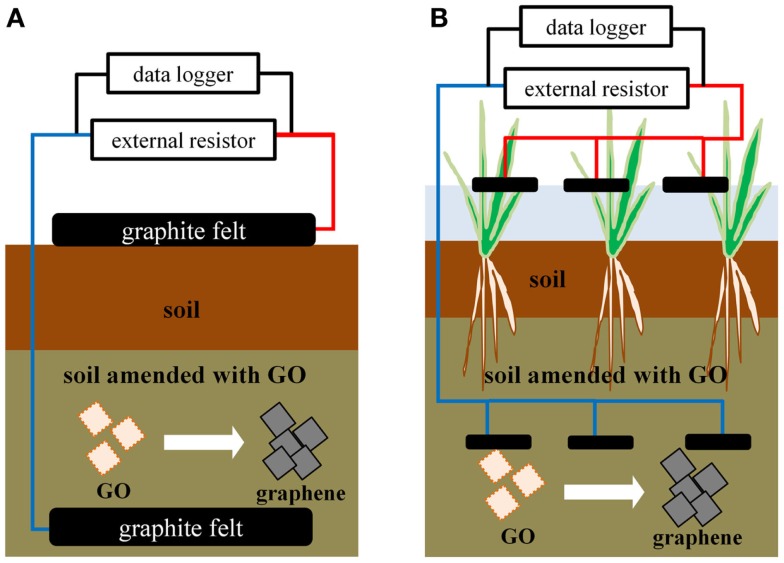
**Structures of the SMFC (A), and PMFC (B), with the addition of GO**.

Soil microbial fuel cell was constructed using commercially available garden soil (PROTOLEAF, Tokyo, Japan) with and without added GO (Figure 1A). In all cases, the soil (750 g⋅wet wt., corresponding to 250 g⋅dry wt.) was mixed with sodium acetate (10 mmol⋅kg^−1^ dry wt.), and adjusted to pH 7.0 by mixing with 10 mM MOPS buffer to give a moisture content of 60–70%. A portion of this mixture (450 g⋅wet wt.) was mixed with GO at the desired concentration ranging from 0.0 to 1.9 g⋅kg^–1^ (dry wt.). An aliquot (150 g⋅wet wt.) of the GO/soil mixture was introduced to a glass container having a working volume of 1000 mL, covered with a graphite felt anode (90-mm diameter, 5-mm thickness, Keego Technologies, Stanford, CA), and then covered with the remainder of the GO/soil mixture. Finally, a portion of the soil mixture that did not contain GO (300 g) was added to the top of the GO/soil mixture in the glass container. The glass container was sealed and incubated at 28°C for 10 days. After incubation, the soil was covered with a graphite felt cathode, and polarized at 28°C for 5 days by connecting the anode and cathode via an external resistor of 1000 Ω. The voltage in the SMFCs was recorded every 60 min using a data logger (T&D Corporation, Nagano, Japan).

### Fed-batch operation

To evaluate GO-enhanced effects, electricity generation was studied in triple fed-batch experiments of SMFC with or without GO (1.0 g⋅kg^–1^), which were constructed as described above. The SMFCs were spiked with 5 mM acetate solution (20 mL) and polarized at 28°C for 5 days by connecting the anode and the cathode via an external resistor of 100 Ω. The acetate solution (40 mL each) was periodically added during the start-up operation of the SMFC. Differences in the surface power density between the SMFC and GO-SMFC samples were statistically analyzed using an unpaired two-tailed *t*-test with Welch’s correction where *p* < 0.05.

### Cyclic voltammetry

Cyclic voltammetry (CV) was performed using an EC stat-300 (EC Frontier, Inc., Kyoto, Japan). The graphite felt anode in the soil was kept as the working electrode, and the graphite felt cathode on the soil surface was used as the counter electrode. An Ag/AgCl electrode was used as a reference electrode and placed in the soil near the cathode. Potentials ranging from −0.8 to +0.2 V (vs. Ag/AgCl) were applied at a scan rate of 0.2 mV⋅ s^−1^ with continuous monitoring of the current response.

### PMFC construction and operation

For construction of the PMFC, seedlings of rice *Oryza sativa* spp. *japonica*, cultivar Koshihirari, were used. These seedlings were obtained from the Japan Agricultural Cooperatives (Miyoshi, Japan) and had an average height of 15 cm. A mixture of garden soil (10 kg⋅wet wt.) with GO (1.0 g⋅kg^−1^ dry wt.) was added to a plastic bucket having a working volume of 15 L (Figure 1B). Three sheets of graphite felt were then placed on the soil surface, and covered with the remainder of the GO/soil mixture, along with 5.0 kg⋅wet wt of garden soil. The bucket was filled with tap water and incubated in an outdoor greenhouse. After 10 days of incubation, three rice seedlings were transplanted into the bucket, and three sheets of graphite felt cathode were floated on the water surface. The cathodes were connected to the anode *via* an external resistor of 22–1000 Ω. The voltage across the resistor was monitored every 60 min using a data logger. The GO-containing PMFCs (GO-PMFCs) were polarized for 82 days. In addition to the GO-PMFC, PMFCs using soils without GO were also prepared using the same procedure to allow comparison of their respective performances.

### Cell counting by microscopy

Microbial biomass in the soil and the MFC was estimated using epifluorescence microscopy. Approximately, 0.5 g of the desired sample was taken into a glass test tube, vortexed for 1 min, diluted with filter-sterilized phosphate-buffered saline solution (pH 7.0), and used for direct cell counting. Samples were stained with 4’,6-diamidino-2-phenylindole (DAPI), mounted with the ProLong^®^ Gold Antifade reagent (Life Technologies, Gaithersburg, MD, USA), and observed under an Olympus model BX-50 phase-contrast/epifluorescence microscope equipped with an Olympus DP70 digital camera (Olympus Corporation, Tokyo, Japan) as described (Yoshida et al., 2006).

## Results

### Reduction of GO and microbial biomass in soil

During the 10 days of incubation, the GO-containing soil turned from brown to black in color, suggesting that the brown-colored GO was reduced to black-colored graphene in the SMFC. No color change was observed in the control soil without GO. The reduction of GO in the soil samples was analyzed by X-ray photoelectron spectroscopy (XPS), according to previous studies (Salas et al., 2012; Tanizawa et al., 2012). However, our attempts to detect the reduced GO were unsuccessful due to the presence of substantial amounts of organic impurities from the soils (data not shown).

Epifluorescence microscopy with DAPI staining showed that the total cell counts in soils with and without GO were 2.8 and 2.4 × 10^9^ cells⋅g^−1^ (dry wt.), respectively. These cell numbers did not change drastically during the overall period of operation.

### Effects of GO on the SMFC

**Figure 2:**
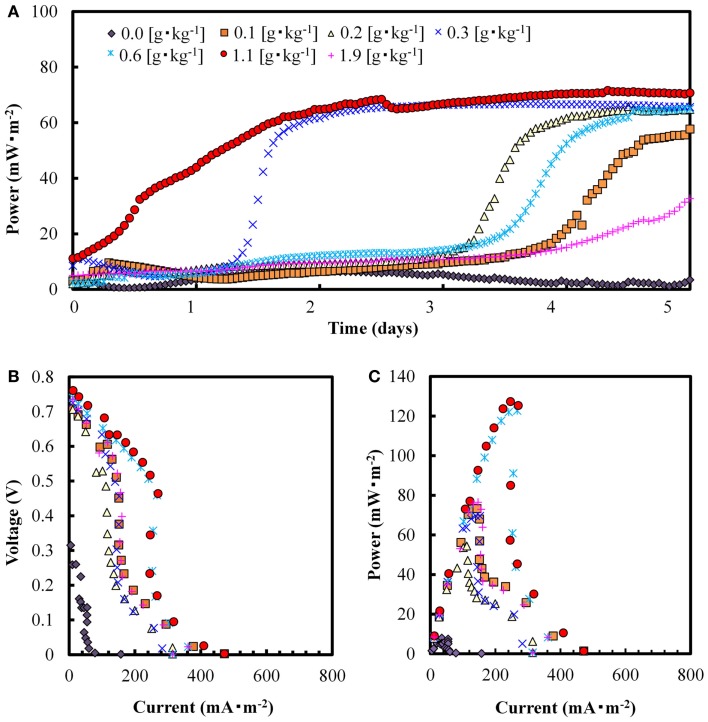
**Electricity production over a period of 5 days (A), voltage vs. current (B) and power density curves (C) on day 7, recorded from SMFCs containing different GO concentrations**.

Figure 2A shows changes in electricity generation in the SMFCs with different concentrations of added GO during 5 days of polarization (1000 Ω). Electricity was generated at higher rates and amounts with added GO than without GO, but the power and velocity were not correlated with its concentrations. The highest surface power density (65 mW⋅m^−2^) was obtained at 1.1 g-GO⋅ kg^−1^ (dry wt.) on day 2. Figures 2B,C show the polarization curve and maximum power density (P_max_) obtained from the SMFCs on day 7. The P_max_ recorded for the GO-SMFCs ranged from 54 to 130 mW⋅m^−2^, while the control without GO produced only 7 mW⋅m^-2^. The P_max_ varied with the concentration of GO, indicating a tendency to enhancement of electricity production by GO. The highest P_max_ value was obtained at a GO concentration of 1.1 g⋅kg^−1^ (dry wt.), which is 16 times greater than the P_max_ value recorded for the GO-free SMFC. At a lower GO concentration (e.g., 0.6 g⋅kg^−1^ dry wt.), however, the power output did not appear to change depending upon its level. This may be due to competition for electron recovery by the microbially reduced GO and the unreduced GO.

The total cell numbers in the SMFCs, as measured by DAPI staining, were in the order of 10^9^ cells⋅g^−1^ (dry wt.) in all cases (Table 1). These results suggest that neither the addition of GO, nor the polarization, exerted significant effects on the total biomass present in the SMFCs.

**Table 1:** **Direct total microbial counts in SMFCs with and without GO**.

Time (days)	Total count [cells⋅g^–1^ (dry wt.)]	
	With GO	Without GO
0	2.8 × 10^9^	2.4 × 10^9^
10	1.5 × 10^9^	3.1 × 10^9^
5 (after polarization)	5.5 × 10^9^	2.1 × 10^9^

*The data show the average cell numbers from measurements taken in duplicate*.

### Fed-batch operation

**Figure 3:**
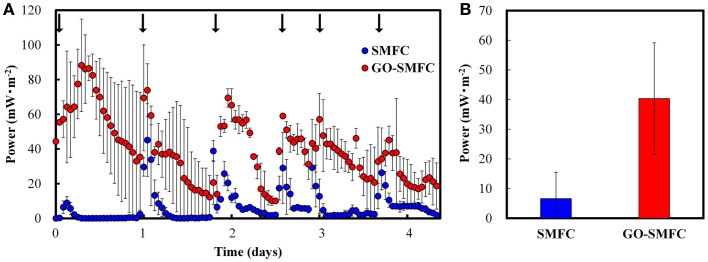
**Electricity production in fed-batch operations over a period of 5 days (A), and the overall average (B), recorded from SMFCs and GO-SMFCs**. The data were measured in triple parallel tests. Arrows indicate addition of acetate.

Electricity generation in SMFCs and GO-SMFCs were evaluated in triple parallel tests using an external resistor of 100 Ω, to give the maximum power densities in the batch experiments (Figure 2C). The GO-SMFC containing 1.0 g⋅kg^−1^ (dry wt.) of GO was evaluated as a representative example of GO-SMFC. Figure 3A shows the average values of electricity generation in each of the triple SMFCs and GO-SMFCs over 5 days of polarization. In this experiment, electricity generation decreased within 1 day. HPLC experiments showed that the initial concentration of added acetate (3.5 mmol⋅kg^−1^) in the soil decreased to below the detection limit within 1 day. Therefore, the SMFCs were periodically supplied with acetate during the experiment.

Generally, electricity was produced at higher rates and amounts with GO than without GO as observed in the batch experiments. The overall average amounts of electricity generated in the GO-SMFCs and SMFCs were 40 ± 19 and 6.6 ± 8.9 mW⋅m^−2^, respectively, and this difference between the two was statistically significant (*p* < 0.01) (Figure 3B). The highest surface power density (88 ± 7.1 mW⋅m^−2^) was obtained with GO-SMFC after 7 h. In SMFC, the highest surface power density achieved was 45 ± 21 mW⋅m^−2^, as recorded after 25 h. This difference in the highest value between GO-SMFC and SMFC was not statistically significant (*p* > 0.05).

### Effects of GO on cyclic voltammetry curves

**Figure 4:**
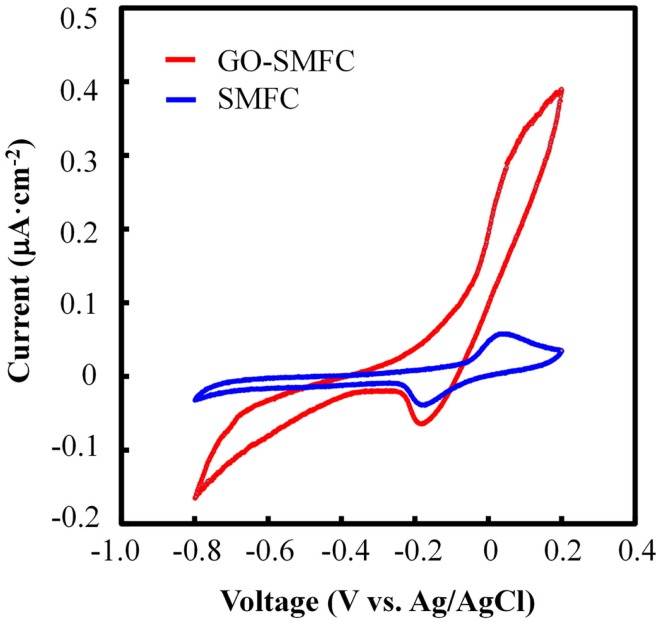
**CV scans of SMFC and GO-SMFCs**.

In order to understand the mechanism by which GO facilitated the generation of electricity in SMFCs, CV analysis was conducted for SMFC and GO-SMFC (Figure 4). In both SMFCs, a pair of redox signals was observed at −63 mV (vs. Ag/AgCl), indicating electron transfer activity *via* a mediator in both SMFC and GO-SMFC. The CV curve of SMFC containing no GO displayed a typical parallelogram shape due to the electric double layer on the graphite anode. In contrast, the shape of the CV curve of GO-SMFC was near sigmoidal, and showed a higher catalytic current than SMFC alone. More specifically, the apparent increase in catalytic current at the oxidative potential suggested that electron transfer in GO-SMFC was promoted. Focusing on the slope of the Δ*E*/Δ*I* curve in the region containing no catalytic reaction, it could be seen that the internal resistance observed for GO-SMFC was smaller than that of SMFC alone. Therefore, in addition to an increase in the catalytic current, a decrease in the internal resistance also contributed to the enhancement of electricity generation in GO-SMFCs.

### The effect of GO addition on the PMFC

**Figure 5:**
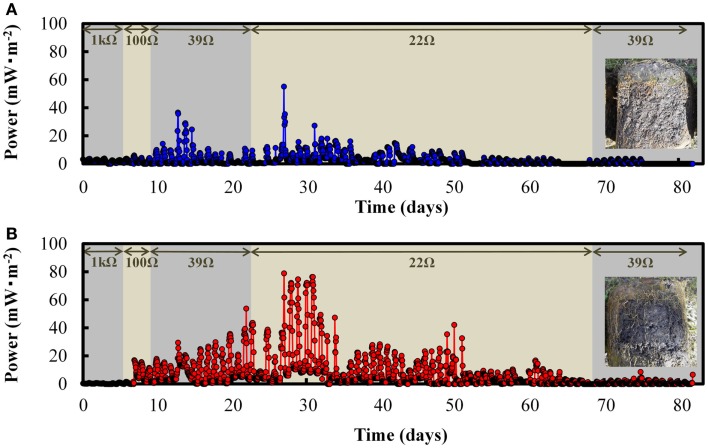
**Electricity generation from the plant microbial fuel cell (PMFC) (A) and a GO-PMFC (B) during the period of polarization**. The photograph insets in **(A,B)** show cross sections of the soil compartments of PMFC and GO-PMFC, respectively.

**Figure 6:**
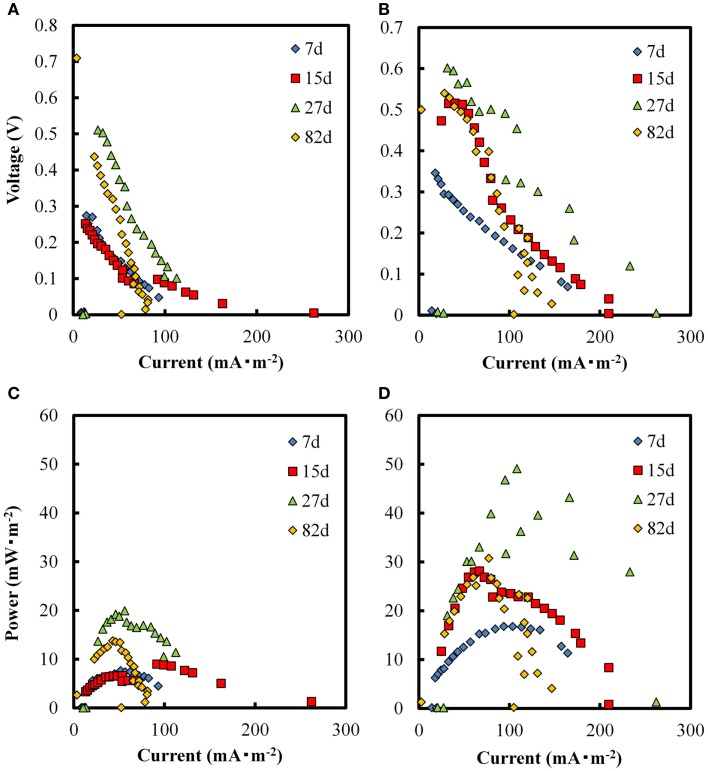
**Voltage vs. current (A), and power density curves (C), from the PMFCs in absence of GO, and voltage vs. current (B), and power density curves (D), from the GO-PMFCs on days 7, 15, 27, and 82 after polarization started**.

We further examined the effects of GO addition on electricity production in PMFC. Based on the results obtained with the SMFC, PMFC was supplied with 1.0 g⋅kg^−1^ (dry wt.) of GO and incubated for 7 days. Figure 5 shows the electricity production in both the GO-PMFCs, and PMFC alone after planting of the seedlings. Electricity production appeared to begin immediately after polarization. In both PMFCs, the production of electricity correlated directly with photosynthetic activity during the daytime. Additionally, both GO-PMFC and PMFC increased electricity production after polarization until day 27 and then decreased gradually. The overall trend was correlated with plant growth and the declining of rice plant vitality. As shown in Figure 6, the P_max_ ranges obtained with PMFC and GO-PMFC were 7.7–20 and 17–49 mW⋅m^−2^, respectively. Also, GO-PMFC produced a higher P_max_ than PMFC during the overall period of operation. Therefore, it is logical to conclude that GO enhanced electricity production in PMFC as well.

Cross section photographs of the soil in both PMFC and GO-PMFC are shown in Figures 5A,B, respectively. The soil was blacker in GO-PMFC than in PMFC. This difference in soil appearance is likely due to the reduction of GO to graphene in the GO-PMFC, as was the case for GO-SMFC. These results provide circumstantial evidence that the soil used in these studies contained microbial populations capable of reducing GO using organic substrates available in the soil or excreted from the plant. Additionally, it appears that the microbially reduced GO was retained in the soil after 82 days of operation. The growth of plants in the two PMFCs and the control pod were similar after 82 days of incubation. In GO-PMFC, the numbers of stalks and leaves counted were 75 and 320, respectively, with the average height of leaves being 81 cm. On the other hand, GO-free PMFC gave 70 of stalks, 290 of leaves, and 84 cm of the average height of leaves. The grain weights obtained from three seedlings were 91 g for GO-PMFC and 92 g for PMFC. These results suggested that GO exerted little or no effects on plant growth in the PMFCs studied.

## Discussion

Previously, GO has been used as an anode modifier in a single-chambered MFC (Yuan et al., 2012). Before this study, however, no information was available on the reduction of GO in soil, or on the enhancement of electricity production in SMFCs and PMFCs. This study has demonstrated that electricity production in both SMFCs and PMFCs is facilitated along with reduction of GO added.

To date, a wide variety of materials and configurations have been investigated as potential anode systems for SMFCs and PMFCs. These systems include graphite rods (Reimers et al., 2006), graphite disks (Tender et al., 2002), graphite felt (Hong et al., 2009; Takanezawa et al., 2010; Song et al., 2012), stainless steel sheets (Dumas et al., 2008), carbon granules (Arends et al., 2012), and activated carbon fiber felt (Song et al., 2012). Focusing on these simply configured MFCs without a separator, catalyst or mediators, similar to those used in this study, electrical power generated in a range of 1.3–27 mW⋅m^−2^ has been reported. The power production in our GO-containing SMFC and PMFC systems showed comparatively higher values than that obtained with previously reported anode materials. Although the exact evaluation of different anode materials for electricity production awaits further study with SMFCs and PMFCs of the same configuration, we discuss the usefulness of our GO-added systems compared to previously reported systems below.

Using a graphite felt cathode, an activated carbon felt showed 3.8-fold higher electricity generation than a graphite felt anode (Song et al., 2012). Karra et al. (2014) constructed a SMFC using an activated carbon cathode and three different anode materials, namely carbon brash (CB), activated carbon nanofiber (ACNF), and granular active carbon (GAC). A variation in electrical output was observed between the three different anode materials, with a higher electricity output being reported in the order of CB > ACNF > GAC. The specific effects of CB and ACNF compared to GAC were 4.5 and 3.1, respectively. Lu et al. (2014) constructed a biochemical system for petroleum hydrocarbon removal, using carbon cloth carrying a Pt/C catalyst, and observed almost a twofold increase in electricity generation where a carbon cloth anode was used in place of a biochar anode. Considering the effects of GO in both SMFC and PMFC together with the effects of other anode materials, the incorporation of GO appears to be one of the promising options for increasing electricity recovery in SMFCs as reported here. Another possible advantage of GO over other anode materials is its flexibility, which allows it to be combined with anodes present in the anolyte. Although positive effects of GO incorporation on electricity generation can be expected, its cost effectiveness should also be taken into account. Commercially available GO is a single-layered material and is expensive at present. In the meantime, GO can be replaced by graphite oxide, which is a bulk material composed of both single- and multi-layered materials.

In this study, we observed that that addition of GO increases catalytic current in SMFC, as shown in CV analysis (Figure 4). In PMFC, the electricity production correlated with sunlight and plant vitality (Figure 5), regardless of whether GO was present. Similar results were obtained with a number of previous studies (Kaku et al., 2008; Strik et al., 2008; Timmers et al., 2013). Generally, GO enhanced electricity production at a higher rate during the daytime than in the night in the case of vital rice, or with matured rice. For example, the daytime electricity output after 27–34 days of plantation was 3- to 66-fold higher with GO than without GO, while only a 0.5- to 7-fold higher output with GO was observed in the night (*p* < 0.01). These results suggest that the enhancement of electricity generation in PMFCs in the presence of GO was mainly caused by enhanced electron transfer with photosynthetic products as the electron donors excreted into the soil mixture.

Stoller et al. (2008) reported that biologically reduced GO had similar conductivity to chemically reduced GO. Both reduced forms of GO increased the electrostatic capacity of the anode by forming a coating on the anode surface (Stoller et al., 2008; Wang et al., 2011). This was caused by increasing the surface area of the electrode through addition of the reduced GO to the surface. Yuan et al. (2012) reported that biologically reduced GO increased the electron transfer rate between the electrode and the microorganisms present, possibly due to an increase in the population of exoelectrogens in the MFC. In our study, no significant relationship between GO addition and the total biomass in the SMFC was observed, possibly because the population density of the present exoelectrogens was not high enough to affect the total population in our SMFC. In a concurrent study, we have identified possible exoelectrogens in SMFC and PMFC with or without GO, and will report these results in elsewhere.

## Conclusion

In view of our results, together with previously reported observations, we can conclude that the electricity generation in SMFCs and PMFCs is enhanced by the reduction of GO added, rather than by GO itself. In addition, the reduced GO likely facilitates electron transfer from oxidization of available substrates to anode in the soil.

To the best of our knowledge, this study is the first to report enhanced electricity production in SMFCs and PMFCs by the addition of GO. This novel process may be applicable for electrical recovery from a wide range of anoxic soils and sediments.

## Author Contributions

YG performed a number of SMFC experiments and drafted the manuscript. YU also performed SMFC experiments. NY carried out the PMFC experiments, electrochemical analysis, and revised the manuscript. TY, RT, and AH participated in data interpretation and revised the manuscript.

## Conflict of Interest Statement

The authors declare that the research was conducted in the absence of any commercial or financial relationships that could be construed as a potential conflict of interest.
